# Synaptic vesicle architecture modulates α-synuclein conformation and pathogenic transitions in Parkinson’s disease

**DOI:** 10.1042/BSR20260364

**Published:** 2026-06-15

**Authors:** Priyatosh Ranjan

**Affiliations:** School of Sciences, Department of Biotechnology, Woxsen University, Hyderabad 502345, Telangana, India

**Keywords:** amyloid, intrinsically disordered proteins, random coil-helix transition, SNARE complex assembly, synaptic vesicle, synucleinopathies

## Abstract

α-Synuclein (α-Syn) is a presynaptic protein implicated in the regulation of synaptic vesicle (SV) organization, soluble N-ethylmaleimide-sensitive factor attachment protein receptor (SNARE) complex assembly, and neurotransmitter release through reversible interactions with curved lipid membranes. Increasing evidence indicates that α-Syn functions as a curvature-sensitive membrane adaptor whose N-terminal amphipathic helices selectively recognize the nanoscale architecture of SVs, thereby promoting vesicle tethering, clustering, and membrane fusion dynamics essential for synaptic transmission. Recent structural and biophysical studies demonstrate that SVs act as catalytic platforms in which membrane curvature, lipid packing defects, molecular crowding, and local physicochemical conditions determine whether α-Syn remains membrane-bound or transitions into pathogenic assemblies. Post-translational modifications, including phosphorylation, nitration, acetylation, and ubiquitination, dynamically regulate α-Syn conformation, membrane engagement, proteostatic turnover, aggregation propensity, and intracellular localization by modulating its structural plasticity and interactions with synaptic membranes. These modifications can alter electrostatic interactions, destabilize amphipathic helices, and shift α-Syn from functional membrane-associated states toward soluble oligomeric and amyloidogenic species. Aging further exacerbates these transitions through alterations in SV lipid composition, membrane fluidity, oxidative membrane damage, impaired proteostasis, and defective vesicle trafficking, thereby destabilizing α-Syn–membrane interactions. The present review discusses how membrane remodeling, vesicle lipid composition, post-translational modifications, protein conformational dynamics, and aging collectively regulate α-Syn phase behavior, membrane binding, and pathological aggregation at the presynaptic terminal. Integrating structural biology, lipidomics, proteomics, and live-cell imaging approaches will identify mechanistic transitions linking physiological membrane engagement to neurodegenerative synucleinopathies and reveal therapeutic opportunities for preventing synaptic dysfunction, toxic condensate formation, and neurodegeneration in Parkinson’s disease and related disorders associated with pathological α-Syn aggregation.

## Introduction

α-Synuclein (α-Syn) is a 140 amino acid presynaptic protein that functions as a key modulator of synaptic vesicle (SV) trafficking and neurotransmitter release in dopaminergic neurons [1–3]. It exists in a dynamic conformational equilibrium between an SV-associated α-helical state and a soluble, intrinsically disordered cytosolic state [4–7]. Through these interactions, it orchestrates vesicle clustering, docking, and priming at the presynaptic terminal. It modulates the assembly of the soluble N-ethylmaleimide-sensitive factor attachment protein receptor (SNARE) complex [8–11]. In doing so, it facilitates rapid vesicle fusion with the presynaptic plasma membrane and ensures efficient neurotransmission and synaptic plasticity [8–11]. Under pathological conditions such as Parkinson’s disease (PD), dementia with Lewy bodies (DLB), multiple system atrophy (MSA), and other synucleinopathies, α-Syn undergoes structural misfolding [12–15]. It then assembles in a nucleation-dependent manner into β-sheet-rich oligomers and fibrillar aggregates known as Lewy bodies (LBs) or Lewy neurites (LNs) [12–15]. These aggregates, characteristic of amyloid fibrils, form elongated supramolecular assemblies stabilized predominantly by non-covalent intermolecular interactions. Although such interactions render amyloid formation intrinsically reversible under defined conditions, the dynamic balance between assembly and disassembly is perturbed in disease [16]. As a consequence, α-Syn aggregates disrupt vesicle trafficking and destabilize SNARE complex function [17]. They also impair Ca^2+^-evoked exocytosis [17]. As a result, α-Syn shifts from a physiological facilitator of vesicle dynamics to a central driver of synaptic failure and neurodegeneration. The capacity for regulated assembly and disassembly is fundamental to proteostasis in the healthy state, and its dysregulation underlies pathological aggregation.

**Figure 1:**
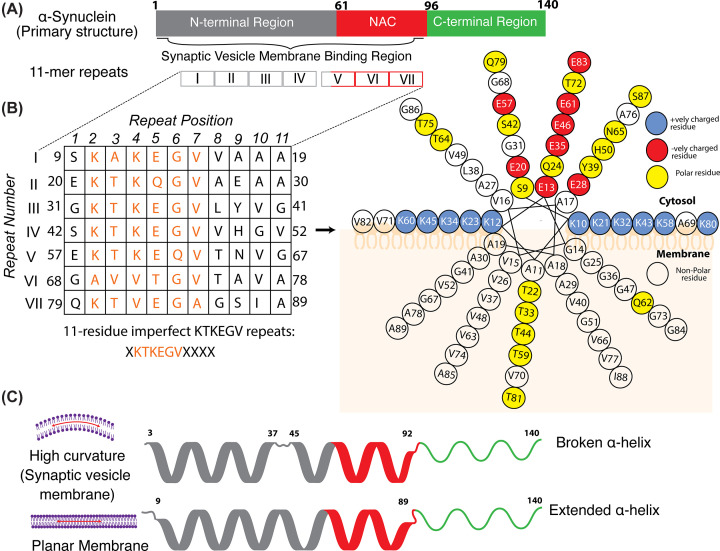
Domain architecture and curvature-dependent helical conformations of α-Synuclein (**A**) Domain-wise organization of monomeric α-Syn. The SV binding N-terminal region of α-Syn is composed of seven imperfect 11-residue KTKEGV repeats, represented by the consensus pattern XKTKEGVXXXX. (**B**) The amino acid sequences corresponding to each 11-residue repeat are shown, with close variants of the KTKEGV motif highlighted in orange. When mapped onto a helical wheel with a periodicity of 3.67 residues per turn, hydrophobic residues segregate to the membrane-facing surface, while polar and charged residues predominantly orient toward the cytosol. (**C**) Upon binding highly curved SV membranes, the lipid binding region of α-Syn forms a broken amphipathic helix. In contrast, it adopts a more continuous, extended α-helical conformation on planar membranes. Schematic representation adapted from Refs. [7],[24], and [25] with additional mechanistic features incorporated.

Structurally, α-Syn is an intrinsically disordered protein that comprises three distinct regions with defined biophysical properties [2] (Figure 1A). The N-terminal segment (residues 1–60) contains amphipathic repeats enriched in both charged and hydrophobic residues, enabling reversible membrane binding [5] (Figure 1B**)**. The central hydrophobic non-amyloid-β component (NAC) region (residues 61–95) constitutes the aggregation-prone core that nucleates amyloid fibril formation [18]. The acidic C-terminal region (residues 96–140) is enriched in negatively charged residues and contains five proline residues that impart structural flexibility and maintain solubility under physiological conditions [19,20]. Together, these domains confer a delicate balance between functional plasticity and aggregation propensity, which becomes dysregulated in disease states. Upon interaction with lipid membranes, the protein’s N-terminal region along with NAC (residues 1–95) (Figure 1A) undergoes a conformational transition to form an α-helical structure [21] (Figure 1C). Notably, every pathogenic missense mutation identified to date maps to the lipid-binding region of α-Syn [22,23].

This region consists of seven 11-residue imperfect amphipathic motifs that align along the membrane surface, positioning their hydrophobic faces toward the lipid acyl chains while orienting polar residues toward the aqueous cytosolic phase [26–28] (Figure 1B**)**. α-Syn’s structural arrangement is tightly governed by membrane curvature and lipid packing density [29,30].

Under certain physicochemical and cellular milieus, α-Syn’s physiological membrane binding transitions into a nucleation-competent state that drives pathogenic self-assembly [31–33]. Upon association with highly curved, anionic lipid bilayers, the protein adopts an α-helical conformation whose metastable nature transiently exposes the hydrophobic NAC domain [34]. This structural plasticity, coupled with local crowding and lateral confinement on vesicle surfaces, promotes nucleation of intermolecular β-sheets [29,34]. Perturbations such as α-Syn familial mutations, lipid compositional imbalance, excessive curvature stress, or oxidative damage further amplify this process, favoring oligomer and fibril formation [35,36]. Thus, SVs act as conditional catalytic platforms, where membrane biophysics dictate whether α-Syn remains a dynamic mediator of vesicle trafficking or becomes a seed for aggregation central to neurodegenerative pathology.

The present review highlights how vesicular membrane biochemistry and curvature coordinate α-Syn binding, balancing its functional role in neurotransmission with its propensity for pathological aggregation. The present review examines how the lipid composition of SVs confers an overall positive curvature that favors α-Syn association through its amphipathic N-terminal helices. The present review further considers how curvature-driven membrane binding enables vesicle clustering and enhances docking and priming at the presynaptic plasma membrane, supporting sustained neurotransmitter release. It also delineates the molecular determinants that bias the equilibrium away from the membrane-bound conformation toward a soluble, conformationally dynamic cytosolic state with heightened susceptibility to aggregation. It addresses how perturbations in lipid composition, membrane curvature, or synuclein concentration (α-, β-, and γ-Syn) can shift these normal interactions toward pathology through membrane-associated or cytosolic aggregation pathways characteristic of synucleinopathies.

## The biochemical architecture of synaptic vesicles: a proteolipid framework for neurotransmission

**Figure 2:**
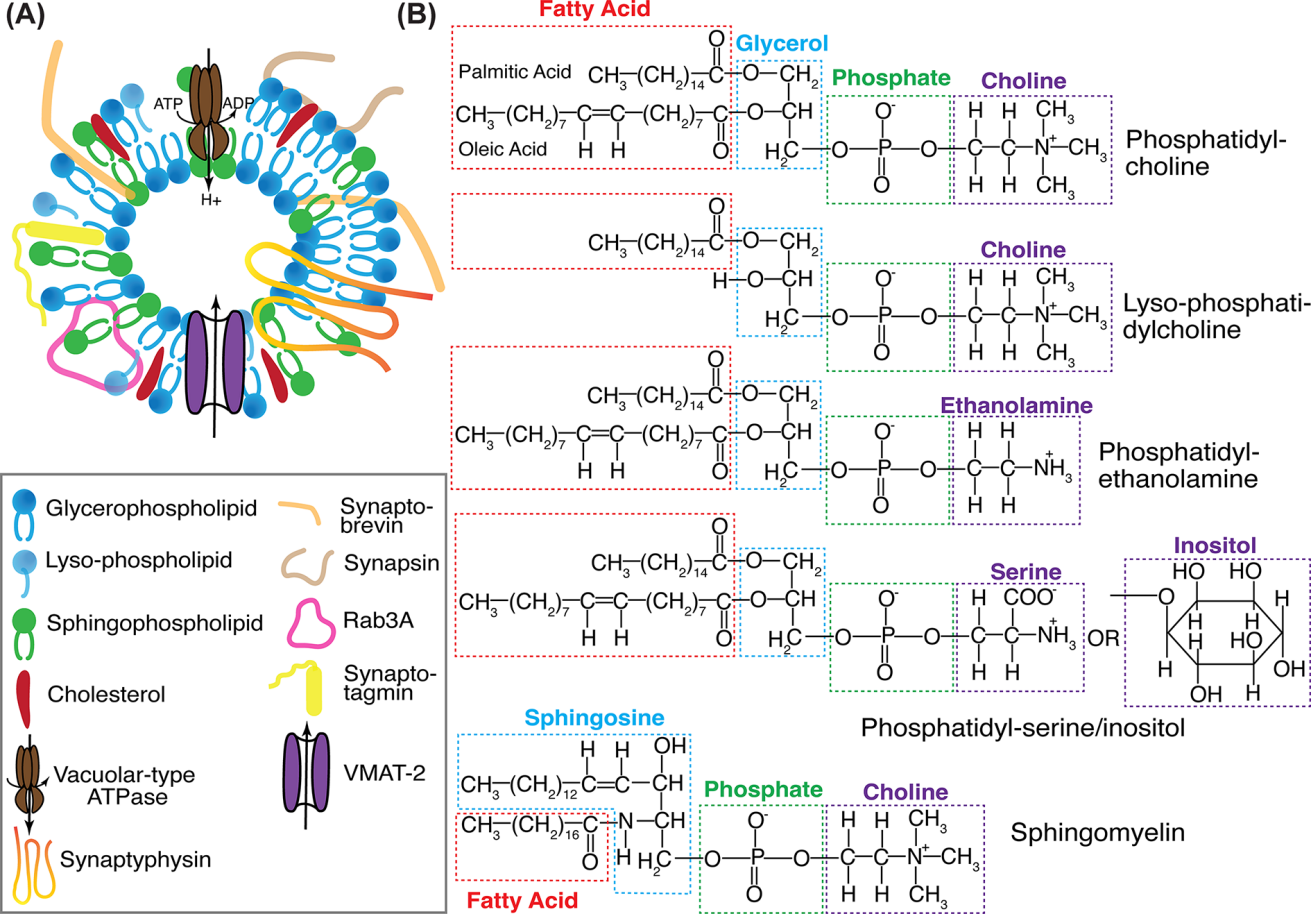
Lipid and protein composition of the SV membrane, highlighting the chemical structures and charge properties of key phospholipids (**A**) Schematic representation of the SV membrane, highlighting key lipid and protein components. (**B**) The chemical structures of representative glycerophospholipids and a sphingophospholipid are shown. Glycerophospholipids such as PC, LysoPC, and PE are zwitterionic and thus electrically neutral at physiological pH, whereas PS and PI carry a net negative charge. Sphingomyelin, a major sphingophospholipid in the membrane, is not synthesized from glycerol but instead contains a sphingosine backbone, an amide-linked fatty acid, and a phosphocholine head group. Schematic representation adapted from Ref. [50] and extended here to incorporate additional features.

SVs are nanoscale spheres (∼40–50 nm in diameter) that encapsulate neurotransmitters, and their extreme curvature is not a passive feature but an emergent property of lipid molecular geometry [37,38]. SVs exhibit a highly specialized lipid architecture composed of a diverse yet precisely balanced mixture of membrane lipids (Figure 2). The vesicular membrane typically contains phosphatidylcholine (PC; ∼17 mol%), phosphatidylserine (PS; ∼6 mol%), phosphatidylethanolamine (PE; ∼20 mol%), phosphatidylinositol (PI; ∼1 mol%), sphingomyelin (∼3.6 mol%), and cholesterol (∼40 mol%) [39,40] (Figure 2A,B). Recent studies show that SVs are enriched in lysophospholipids, particularly lysophosphatidylcholine (LysoPC) [41, 42, 43, 44]. These lipids enhance α-Syn association with the vesicle membrane and also support key functions, including vesicle clustering and the regulation of SV dynamics [41]. This compositional complexity establishes not only the structural integrity of the vesicle but also the biophysical properties that enable its dynamic function [45].

In addition, SVs comprise a highly conserved and specialized set of integral and peripheral membrane proteins that orchestrate neurotransmitter storage, docking, fusion, and recycling (Figure 2A**)**. Central to this machinery are the SNARE proteins, notably vesicle-associated membrane protein 2 (VAMP2; also known as synaptobrevin 2), which form a ternary complex with syntaxin-1 and SNAP-25 on the presynaptic plasma membrane to drive membrane fusion [37,46]. The vesicular proton pump (V-ATPase) establishes the proton motive force required for neurotransmitter accumulation by vesicular transporters such as vesicular monoamine transporter [47,48]. Additional regulatory and scaffolding components including synaptophysin, synaptotagmin, Rab3A, and Rabphilin coordinate vesicle trafficking, calcium sensing, and membrane tethering [37,38,49] (Figure 2A). Collectively, this finely tuned proteolipid network enables SVs to perform the rapid, cyclical exo- and endocytic events fundamental to neuronal signaling.

## Molecular geometry meets neurobiology: how lipid curvature shapes synaptic vesicle architecture and dynamics

**Figure 3:**
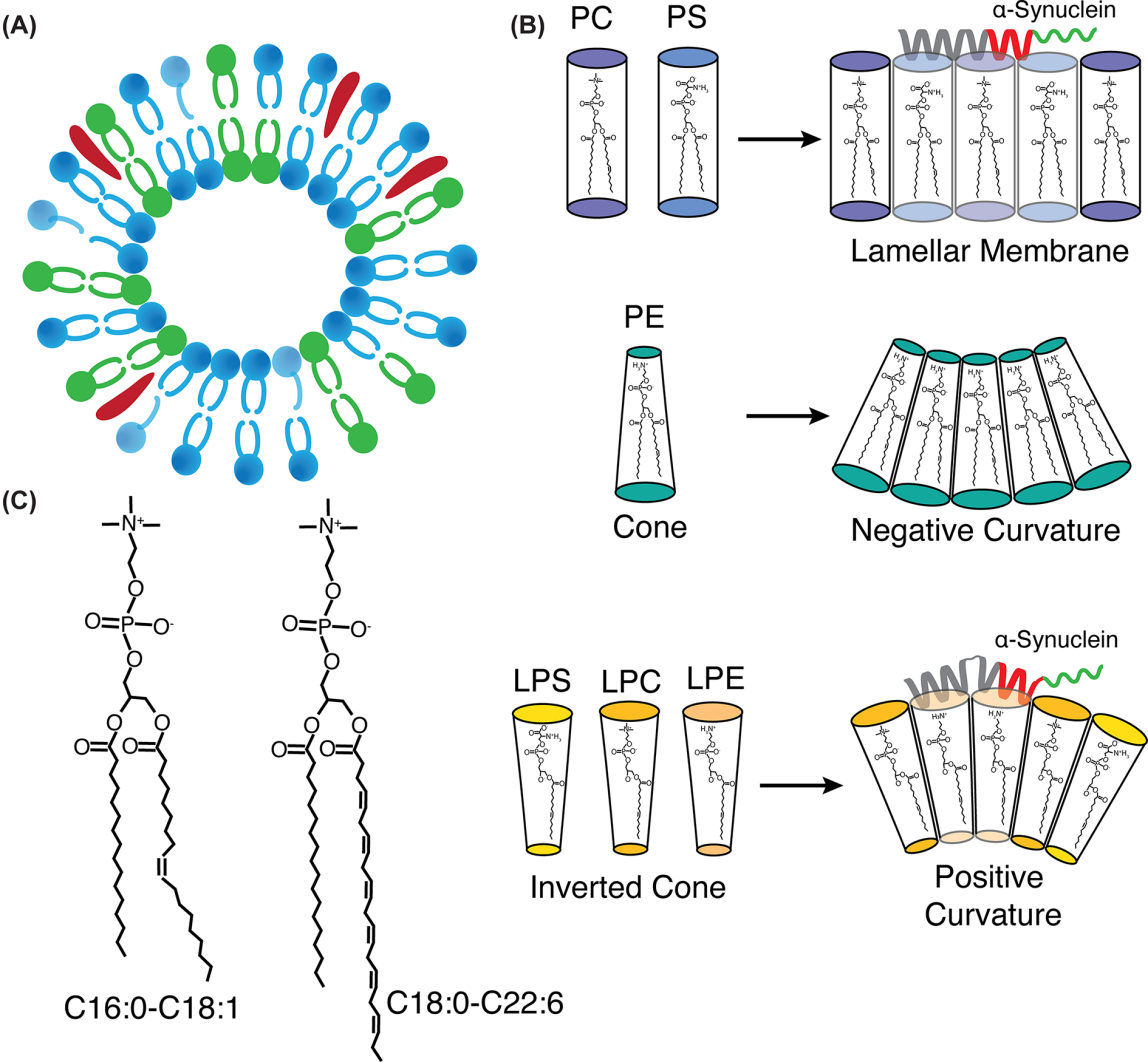
Biophysical basis of membrane curvature driven by SV lipids (**A**) Schematic representation of a SV membrane illustrating its typical high-curvature architecture. The bilayer is composed of glycerophospholipids (blue), sphingophospholipids (green), and cholesterol (red). For simplicity, membrane-associated proteins are not depicted. (**B**) Illustration of distinct curvature types: lamellar (flat), negative (concave), and positive (convex), determined by the relative dimensions of lipid head groups and hydrophobic tails. (**C**) Representative structures of unsaturated fatty acids (mono- and polyunsaturated) are commonly found in SV membranes, highlighting their role in modulating membrane fluidity and curvature. Binding to planar membranes promotes a continuous α-helical structure in α-Syn, whereas positively curved membranes favor a broken helix. Schematic representation adapted from Ref. [56] with minor additions.

The interplay between lipids and embedded proteins modulates membrane curvature, fluidity, and electrostatic surface potential, key determinants of vesicle trafficking, docking, and fusion. Membrane curvature arises from the molecular geometry of constituent lipids, which determines how they pack within the bilayer (Figure 3). PC and PS represent the archetype of a cylindrical lipid that promotes no intrinsic curvature [40,45,51] (Figure 3B). Its large, hydrated head group and two comparably sized acyl chains balance the cross-sectional areas of the polar and hydrophobic regions, resulting in a packing parameter close to unity. This geometric symmetry allows PC and PS molecules to pack in parallel without generating bending stress, stabilizing planar, lamellar membranes [40,45,51]. In contrast, lipids with relatively small head groups and bulky hydrocarbon tails, such as PE, exhibit a cone-like molecular shape. These lipids favor negative curvature, bending the membrane inward and facilitating the formation of highly curved structures such as vesicle necks and fusion intermediates [40,45,51] (Figure 3B).

Building on this geometric framework, SVs are enriched in lipids that intrinsically favor positive curvature (Figure 3B). This property is central to their nanoscale, highly curved architecture and to their dynamic behavior during neurotransmission. Among these lipids, lysophospholipids and PI display an inverted cone-shaped geometry [40,45,51]. They contain a large hydrophilic head group and a narrow hydrophobic tail, yielding a packing parameter of less than one (P < 1). This molecular shape promotes outward bending of the bilayer, stabilizing the convex curvature characteristic of SVs (Figure 3B). The abundance of such curvature-inducing lipids enhances membrane flexibility and fusion competence, supporting efficient vesicle exocytosis and recycling [40,45,51]. Furthermore, spatial enrichment of inverted cone-shaped lipids forms curvature-sensitive microdomains that facilitate the recruitment of proteins like α-Syn and endophilin, thereby coupling lipid geometry to the regulation of synaptic membrane remodeling [1,52,53]. Studies also indicate that long-chain polyunsaturated fatty acids play an essential role in sustaining the efficiency of SV recycling [54,55] (Figure 3C).

The spherical morphology of SVs is encoded by the collective geometry of their lipid constituents, rather than emerging from membrane mechanics alone. The bilayer exhibits pronounced asymmetry, with PE, PI, and PS enriched in the inner (cytosolic) leaflet, and PC and LysoPC predominating on the outer (luminal) side [45,51]. This compositional imbalance generates intrinsic bilayer tension that, together with the vesicle’s nanoscale diameter, imposes a curvature. Such curvature is not merely structural; it is functionally indispensable, facilitating vesicle budding, trafficking, and calcium-triggered fusion. Thus, the molecular shape of lipids encodes curvature as a biophysical language that governs the efficiency of synaptic neurotransmission.

## Curvature dictates conformation: how membrane geometry bends the α-helix of α-synuclein

On the surface of unilamellar vesicles, α-Syn adopts a continuous, extended α-helical conformation spanning residues 9–89 [7,57–59] (Figure 1C). In this configuration, the helix lies parallel to the bilayer, enabling uniform hydrophobic and electrostatic interactions across its amphipathic surface, a geometry optimized for association with relatively flat lamellar membranes. In contrast, on highly curved membranes such as those of SVs (∼40–50 nm in diameter) (Figure 3A,B), α-Syn adopts a bent or ‘broken’ α-helical conformation. Structural analyses using site-directed spin labeling, electron paramagnetic resonance (EPR), and nuclear magnetic resonance (NMR) spectroscopy demonstrate that this state comprises two α-helical segments (residues 1–37 and 45–92) connected by a short flexible linker (residues 38–44) that functions as a curvature-sensitive hinge [60–62]. This conformational adaptability underlies α-Syn’s preferential association with small-diameter vesicles [5,63], contributing to its enrichment on SVs within dopaminergic neurons of the substantia nigra region of the midbrain [1].

Unilamellar vesicles and SVs share a single lipid bilayer architecture, yet they differ fundamentally in biological role and membrane composition. Unilamellar vesicles are experimental model systems that simplify membrane structure to facilitate mechanistic analysis, typically lacking the protein density and lipid heterogeneity found *in vivo*. SVs, in contrast, are specialized neuronal organelles whose membranes are enriched in cholesterol, compositionally asymmetric, and densely populated with proteins essential for neurotransmitter storage, release, and recycling. These intrinsic differences shape membrane behavior and function, limiting the direct translation of observations from synthetic vesicles to physiological synaptic processes.

Fusco et al. conducted a comprehensive structural analysis elucidating how α-Syn engages with lipid membranes [64]. Combining solid-state and solution NMR spectroscopy, the study demonstrates that the N-terminal segment (residues 6–25) forms a membrane-anchored α-helix, while the central region (residues 26–97) functions as a lipid-sensitive membrane sensor that modulates binding strength in a lipid-specific manner. In contrast, the C-terminal tail remains largely disordered and only weakly associated with the bilayer [64]. Collectively, these findings delineate the distinct functional contributions of α-Syn’s three regions to its reversible membrane association and provide mechanistic insight into how perturbations in these interactions may drive pathological aggregation in neurodegenerative disease. Overall, the curvature-dependent folding enables α-Syn to maximize electrostatic and hydrophobic interactions with the outer lipid leaflet while maintaining the conformational plasticity required to adapt to vesicular curvature. This configuration is energetically preferred on small vesicles, where the membrane’s pronounced curvature precludes the accommodation of a single rigid α-helix without inducing steric strain.

## Post-translational modifications modulate α-synuclein membrane interactions and functional regulation

**Figure 4:**
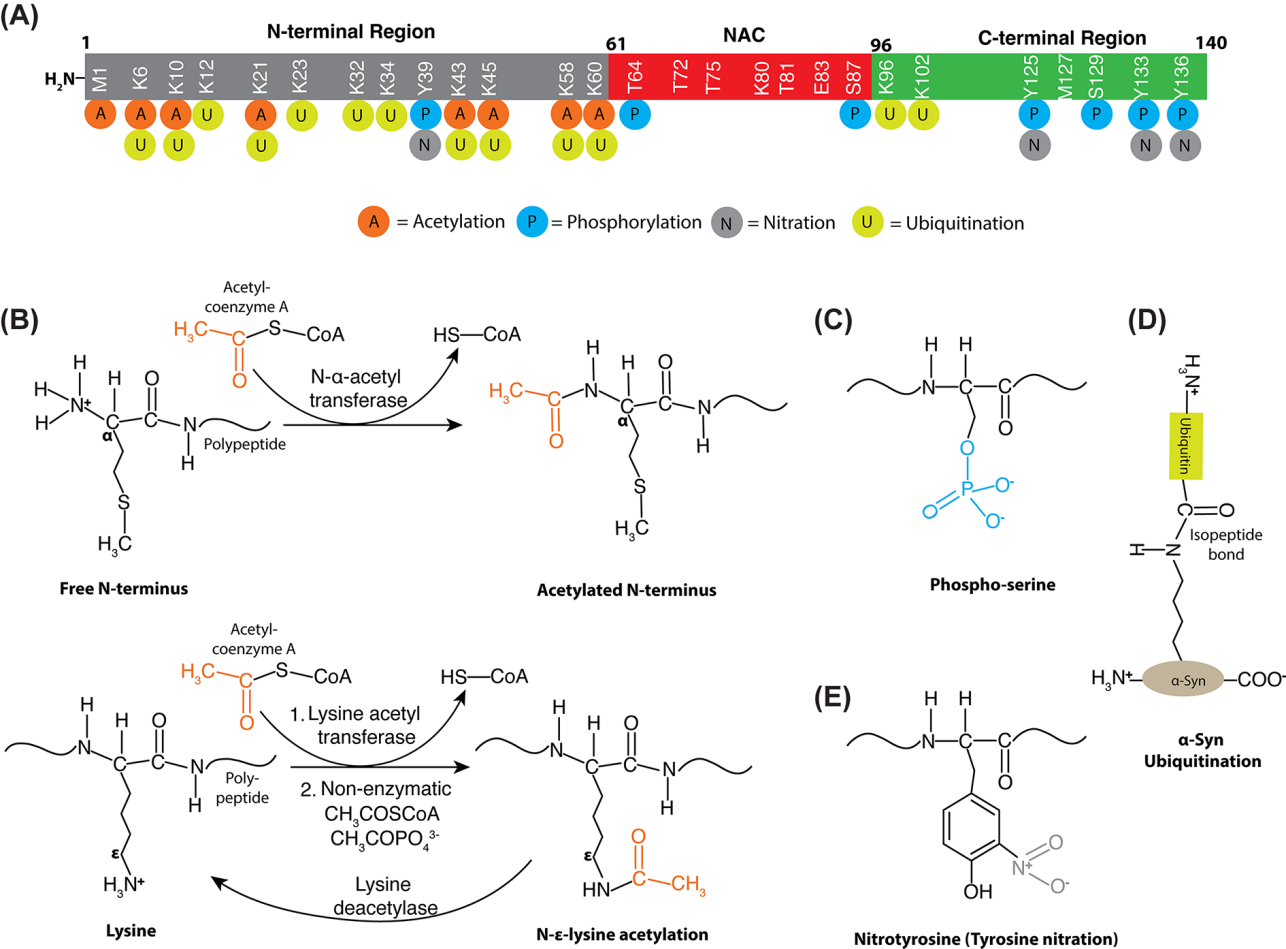
Key PTMs regulating α-Synuclein (**A**) Major PTM sites in α-Syn. Residues susceptible to acetylation, phosphorylation, nitration, and ubiquitination are indicated. (**B**) N-terminal (N-α) acetylation is catalyzed by N-α-acetyltransferases using acetyl-CoA as the acetyl donor. Lysine side-chain acetylation (N-ε-acetylation) is mediated by lysine acetyltransferases or can occur non-enzymatically through acetyl-CoA or acetyl phosphate. Certain acetylation events are reversible and can be removed by lysine deacetylases. (**C**) Phosphorylated serine is generated when a phosphate group is covalently attached to the hydroxyl group of a serine residue. (**D**) Ubiquitin is conjugated to α-Syn through formation of an isopeptide bond between the terminal carboxyl group of Gly76 of ubiquitin and the ε-amino group of a lysine residue within α-Syn. (**E**) Tyrosine nitration modification involves substitution of a hydrogen atom at the 3-position of the tyrosine phenolic ring with a nitro group.

Post-translational modifications (PTMs) provide a dynamic regulatory framework that governs α-Syn conformational plasticity, membrane engagement, and proteostatic fate (Figure 4A). By altering charge distribution, steric constraints, and long-range intramolecular interactions, PTMs modulate the equilibrium between functional membrane-bound states and cytosolic species predisposed to aggregation.

### Acetylation

The process of acetylation involves the addition of an acetyl group (CH_3_CO) to a molecule. Protein acetylation primarily occurs on amino groups, but acetylation of serine and threonine residues has also been reported [65–67]. Acetylation targeting amino groups, present at the protein N-terminus and on basic amino acid side chains, has received the greatest experimental and mechanistic attention. N- α-acetylation is distinct from the broader concept of N-terminal acetylation. N- α-acetylation specifically describes modification of the free α-amino group of the first residue, typically methionine (Figure 4B). In contrast, N-terminal acetylation may refer more generally to acetylation events occurring within the amino-terminal region of a protein composed of multiple residues. In addition to these modifications, acetylation can also occur at the amino group of ε-carbon of lysine side chains (N-ε-acetylation), a reaction catalyzed by a distinct class of enzymes known as lysine acetyltransferases (Figure 4B) [68,69]. The acetyl moiety, supplied by acetyl-coenzyme A, can be enzymatically transferred either co- or post-translationally to the α-amino group at the protein N-terminus or to the ε-amino group of lysine side chains [70]. Under physiological conditions, α-Syn exists predominantly in an N- α-acetylated state. This modification is consistently observed across diverse biological sources, including brain tissue, erythrocytes, and cultured mammalian cell lines [71–74]. N- α-acetylation enhances the association of α-Syn with lipid and SV membranes by increasing its binding efficiency [75,76]. Despite this elevated membrane affinity, structural studies using solid-state NMR indicate that the modification does not significantly alter the conformation adopted by α-Syn in its membrane-bound state [77]. Recent studies on the side-chain acetylation of lysine residues in α-Syn indicate that N-ε-acetylation at K43 impairs membrane binding, with acetylation at K12 producing a similar but comparatively weaker effect [78].

### Phosphorylation

Protein phosphorylation is a reversible PTM in which protein kinases catalyze the covalent transfer of a phosphate group to specific amino acid residues [79]. α-Syn is phosphorylated at multiple sites, including serine, tyrosine, and threonine residues (Figure 4A) [80–82]. Among these, phosphorylation at Ser129 (pS129) represents the predominant modification detected in LBs and LNs (Figure 4C) [83,84]. Increasing evidence, however, indicates that physiological pS129 is also present on monomeric α-Syn at synapses, where it contributes to the fine regulation of synaptic function [85–87]. Accordingly, pS129 likely exerts context-dependent effects, supporting normal α-Syn regulation under physiological conditions while also being linked to the accumulation of insoluble aggregates characteristic of PD [88]. Samuel et al. reported that pS129 exerts only a modest influence on α-Syn membrane binding [89]. Biochemical analyses showed that phosphomimetic mutants retain the ability to interact with negatively charged membranes, indicating preservation of the N-terminal lipid-binding domain [89]. The modification primarily affects conformational dynamics mediated by the acidic C-terminal region rather than directly disrupting membrane attachment. Recent work suggests that pS129 is preferentially associated with membrane-bound α-Syn, rather than with soluble cytosolic populations [86]. Membrane-associated, helical α-Syn appears to be preferentially targeted by polo-like kinase 2, with neuronal activity promoting phosphorylation within synaptic, membrane-enriched regions [86].

These observations suggest that phosphorylation at Ser129 occurs subsequent to, or concomitant with, membrane engagement rather than disrupting membrane association. Instead of driving dissociation, this modification is proposed to modulate the functional properties of α-Syn at presynaptic terminals. Collectively, these findings support a model in which pS129 acts as a regulatory signal that fine-tunes the behavior of membrane-bound α-Syn during synaptic activity. Phosphorylation at Tyr39 (pY39) has been shown to attenuate the lipid-binding capacity of α-Syn. Modification at Y39 reduces membrane association of the segment corresponding to helix-2 within the broken-helix conformation, thereby potentially increasing the availability of this region to engage with other membrane surfaces [90]. Overall, these findings indicate that site-specific phosphorylation differentially regulates α-Syn, with pS129 primarily fine-tuning the function of membrane-bound protein during synaptic activity, whereas modifications such as pY39 selectively remodel membrane interactions and conformational dynamics.

### Ubiquitination

Ubiquitin is an evolutionarily conserved 76-amino acid protein of ∼8.5 kDa that regulates protein fate through covalent conjugation [91,92]. This modification is established via the formation of an isopeptide bond between the terminal carboxyl group of Gly76 of ubiquitin and the ε-amino group of a lysine residue within the substrate (Figure 4D) [93]. Ubiquitination proceeds through an ATP-dependent enzymatic cascade involving three classes of enzymes: the ubiquitin-activating enzyme (E1), which activates ubiquitin; ubiquitin-conjugating enzymes (E2s), which accept the activated ubiquitin; and ubiquitin ligases (E3s), which provide substrate specificity and catalyze ubiquitin transfer to the target protein [93]. Depending on the number of ubiquitin moieties attached, substrates may undergo monoubiquitination, multi-monoubiquitination at multiple lysine residues, or polyubiquitination, in which ubiquitin molecules form chains on a single lysine site [94]. Ubiquitin-dependent proteolysis governs a broad spectrum of cellular processes, including cell cycle progression, DNA repair and transcriptional regulation, protein quality control, and immune signaling [93,95]. Disruption of this tightly controlled degradation pathway contributes directly to the pathogenesis of numerous human diseases. Mass spectrometric analysis in a cell-free system identified several seven in absentia homolog 2 dependent ubiquitination sites on α-Syn, including K10, K12, K21, K23, K34, K43, and K96 (Figure 4A) [96]. Notably, these positions overlap with ubiquitination sites previously mapped in LBs, particularly K12, K21, and K23 [84]. Hejjaoui et al. designed a site-specifically monoubiquitinated α-Syn species at Lys6 to examine how this modification influences membrane interactions [97]. Biophysical analyses demonstrated that monoubiquitination at this N-terminal position does not markedly impair the protein’s ability to adopt a membrane-induced α-helical conformation upon binding to synthetic lipids [97]. Accordingly, the intrinsic lipid affinity of α-Syn is largely preserved despite the presence of a single ubiquitin moiety. However, ubiquitination significantly suppresses fibril formation and stabilizes soluble conformers, thereby potentially shifting the equilibrium between cytosolic and membrane-associated pools [97]. These findings indicate that monoubiquitination does not directly abrogate membrane binding but may indirectly influence membrane engagement through modulation of conformational dynamics and aggregation behavior. Ubiquitination at K45, K58, and K60 may act as a surveillance mechanism that removes potentially harmful α-Syn from membranes and directs it toward intracellular sorting or degradation pathways [98]. Ubiquitinated α-Syn is trafficked into endosomes and targeted to lysosomes for degradation, indicating that ubiquitin serves as a signal for detachment from membrane sites and routing into the degradation machinery [98]. Endogenous ubiquitinated species are observed in LBs and in neuronal models, suggesting that altered membrane engagement and trafficking contribute to pathogenic inclusion formation. Together, these findings support a model in which ubiquitination attenuates α-Syn’s stable membrane binding by tagging membrane-associated pools for intracellular sorting and turnover rather than direct lipid engagement.

### Nitration

Tyrosine nitration is a stress-responsive PTM that commonly arises under conditions of oxidative and nitrosative imbalance. Chemically, it involves the substitution of a hydrogen atom at the third position of the tyrosine phenolic ring with a nitro group (–NO_2_), resulting in the formation of 3-nitrotyrosine (Figure 4E) and altering the residue’s steric and electrostatic properties [99,100]. α-Syn contains four tyrosine residues, Y39 within the N-terminal region and Y125, Y133, and Y136 within the C-terminal region, all of which are susceptible to nitrative modification [101]. Site-specific nitration was shown to allosterically regulate α-Syn membrane interactions by altering long-range intramolecular contacts rather than directly modifying the lipid-binding interface [102]. Studies have demonstrated that nitration, particularly at Tyr39, significantly weakens binding to negatively charged lipid vesicles by destabilizing the membrane-induced α-helical conformation within the N-terminal region [102,103]. Importantly, modifications at distal C-terminal tyrosines (Y125/133/136) also reduced membrane affinity, revealing that oxidative changes propagate through the intrinsically disordered structure to modulate lipid engagement. Biophysical measurements further indicated that nitration shifts the conformational ensemble toward states incompatible with stable membrane association. Overall, the work established oxidative nitration as an allosteric mechanism that diminishes physiological membrane binding of α-Syn and may promote pathogenic redistribution of the protein under oxidative stress conditions [102].

Taken together, these PTMs impose layered regulatory control over α-Syn membrane biology. Constitutive modifications such as N- α-acetylation and activity-coupled pS129 phosphorylation fine-tune membrane engagement and synaptic function, whereas stress-associated nitration and selective ubiquitination promote membrane disengagement, redistribution, or turnover. The dynamic interplay among these modifications likely governs the balance between functional membrane-associated α-Syn and aggregation-prone cytosolic species, thereby linking structural plasticity to neurodegenerative vulnerability.

## α-Synuclein shapes the synaptic vesicle landscape via curvature-dependent multivalent tethering

**Figure 5:**
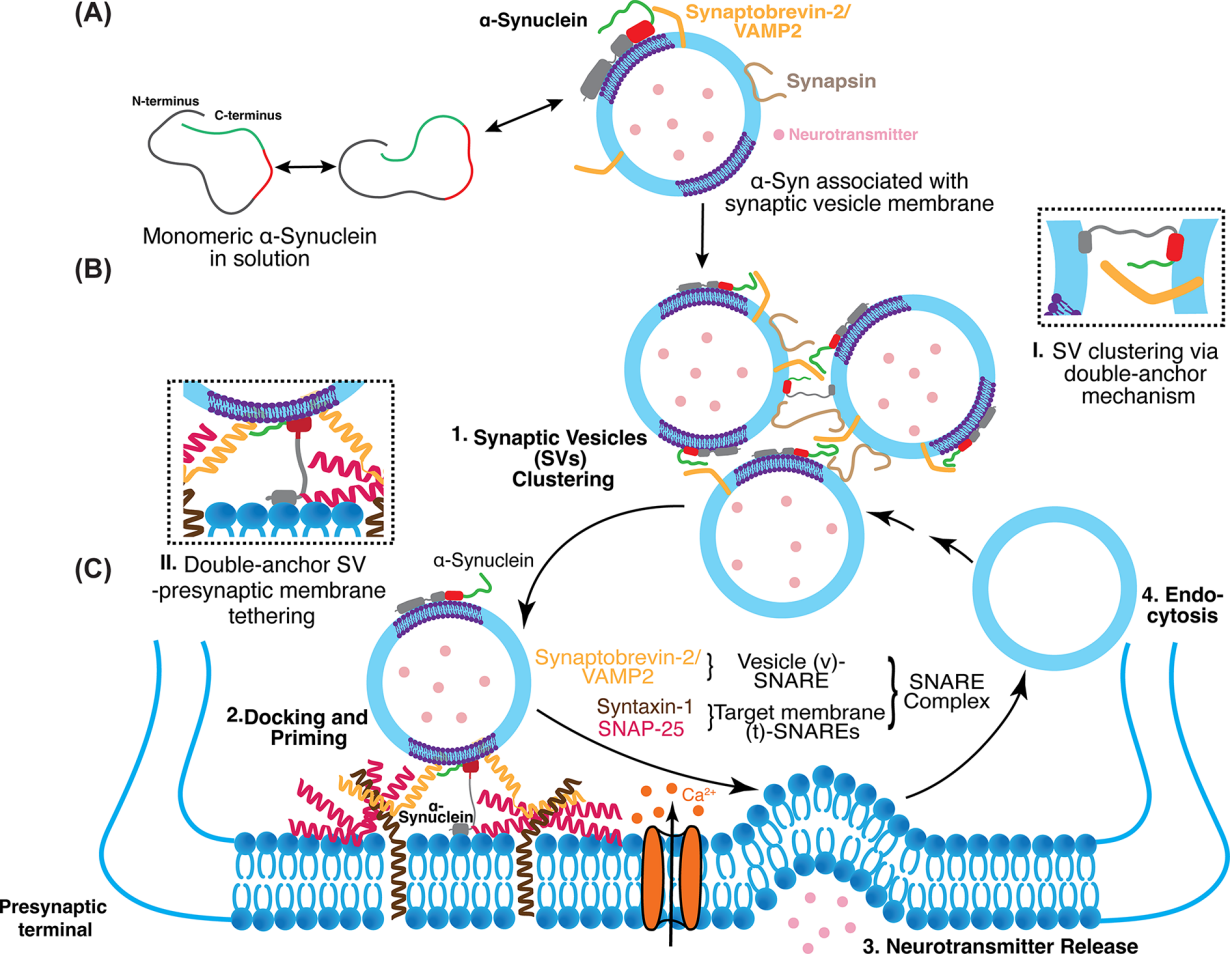
Conformational switching of α-Synuclein drives vesicle clustering and neurotransmitter release (**A**) In solution, α-Syn samples a highly dynamic ensemble of conformations characteristic of intrinsically disordered proteins. Upon binding to SV membranes, it transitions into a helix-rich structure, while its C-terminal region remains unstructured and flexible. Membrane-associated α-Syn also interacts with the vesicle-integral protein, synaptobrevin-2/VAMP2. (**B**) These interactions promote the clustering of SVs at the presynaptic terminal, a process further reinforced by scaffolding proteins such as synapsin. Inset I illustrates a magnified view in which a single α-Syn molecule bridges two adjacent SVs via a double-anchor mechanism. (**C**) During vesicle docking, v-SNARE and t-SNARE proteins assemble into a tight SNARE complex that drives membrane fusion and neurotransmitter release. Inset II provides a zoomed-in view showing a single α-Syn molecule simultaneously engaging both an SV and the presynaptic membrane through a double-anchor mechanism. Schematic representation adapted from Ref. [25] with additional details incorporated.

α-Syn promotes SV clustering by functioning as a curvature-sensitive, multivalent membrane tether that dynamically links adjacent vesicles through low-affinity interactions with both lipids and vesicle-associated proteins [9,41] (Figure 5A,B). Upon binding to SVs, α-Syn influences vesicle fusion by engaging with the SNARE machinery [9,41]. It directly interacts with VAMP2, a key SNARE component, thereby modulating the assembly and activity of the fusion complex [11,104] (Figure 5A,B). Studies by Fusco et al. provide a mechanistic framework for understanding how α-Syn organizes SVs [105]. Using NMR, EPR, cryo-EM, and STED, the study shows that α-Syn promotes SV clustering through a ‘double-anchor’ mechanism, where its N-terminal (residues 1–25) and central (65–97) regions independently tether two vesicles spaced up to ∼150 Å apart [105] (Figure 5B, Inset I). This dynamic architecture underlies its ability to cluster vesicles at presynaptic terminals while maintaining structural adaptability. Pathogenic mutations perturb this organization and drive α-Syn aggregation, illustrating how subtle alterations in its membrane-bound conformation can shift the protein from a normal scaffolding function to pathological self-assembly [22,23].

A recent study by Wang et al. reveals that VAMP2 acts as a molecular chaperone for α-Syn within SV co-condensates [11]. The juxtamembrane domain of VAMP2 directly interacts with the C-terminal region of α-Syn, facilitating the formation of dynamic condensates composed of vesicles, α-Syn, and associated presynaptic proteins [11]. This interaction promotes vesicle clustering and supports SNARE complex assembly while concurrently preventing α-Syn from forming aggregation-prone oligomers and fibrils. By linking α-Syn’s physiological membrane organization with protection against its pathological misfolding, the study uncovers a mechanism through which VAMP2 safeguards presynaptic proteostasis and synaptic function.

Overall, interactions between α-Syn and SV-associated lipids and proteins are central to maintaining precise control over neurotransmitter release. Through a spectrum of transient and reversible contacts, α-Syn associates with several presynaptic partners, including VAMP2 [41], synapsins [106,107], and Rab3a [108,109]. These interactions are primarily mediated by its intrinsically disordered C-terminal tail [11,110], whose conformational flexibility allows α-Syn to adapt to multiple binding partners. By acting as a dynamic molecular scaffold, α-Syn fine-tunes vesicle trafficking, docking, and fusion, thereby integrating synaptic organization with functional plasticity.

## From synaptic scaffolds to vulnerability: synuclein-mediated maintenance of presynaptic integrity

The synaptic fusion pore represents a transient nanoscale conduit connecting the SV to the presynaptic plasma membrane [111]. The SNARE complex plays a central role in docking and fusing SVs with the presynaptic membrane [9] (Figure 5C). Its assembly is driven by the progressive zippering of three core SNARE proteins: VAMP2 on vesicle membranes and syntaxin-1 together with SNAP-25 on the presynaptic membrane [112,113]. This process forms a tightly coiled four-helix bundle that brings the membranes into proximity, enabling fusion (Figure 5C). Calcium influx, mediated by regulatory proteins such as synaptotagmins and complexins, synchronizes this event to ensure rapid neurotransmitter release [112,113]. Beyond the SNARE machinery, α-Syn modulates SV docking in a lipid-dependent manner [114]. Man et al. showed that α-Syn preferentially associates with the inner leaflet of the presynaptic membrane, stabilizing vesicle docking via a double-anchor mechanism: residues 1–25 form an amphipathic helix as the first anchor, while residues 65–97 form a second helical anchor (Figure 5C, Inset II) [114]. Structural analyses reveal that changes in the lipid environment, particularly under pathological conditions, alter α-Syn’s conformation and membrane affinity, with the NAC-overlapping region emerging as a key determinant.

Collectively, α-Syn anchors to SVs through its amphipathic helix while engaging vesicle-associated proteins via multivalent, low-affinity interactions. Its acidic, flexible C-terminal region provides additional transient cytosolic contacts, enabling vesicle clustering, docking stabilization, and fine-tuning of release probability. The same structural plasticity that supports these physiological functions also creates vulnerability to misfolding and aggregation under disease-relevant conditions.

In addition to α-Syn, other abundantly expressed neuronal proteins, namely β- and γ-synuclein [4], have also been examined for their contributions to neuronal function. Jacqueline Burré and colleagues demonstrated that sustained presynaptic SNARE-complex formation depends on a specialized chaperone-like function provided by synucleins [9]. Their work showed that mice lacking α-, β-, and γ-synucleins developed progressive neurological deficits with aging, accompanied by reduced SNARE-complex levels and shortened lifespan. These observations suggest that synucleins play an important role in maintaining presynaptic stability by sustaining SNARE assembly and synaptic efficiency over time. Another study by Becket Greten-Harrison et al. demonstrated that synucleins are primarily required for maintaining synaptic organization and long-term neuronal function rather than for early neuronal development [115]. Simultaneous deletion of α-, β-, and γ-synucleins resulted in smaller presynaptic terminals and progressive impairment of synaptic transmission that became more evident with aging. Although young knockout mice initially showed compensatory adaptation, this resilience declined over time, leading to functional deterioration. The deficits were mainly linked to altered synaptic architecture and axonal integrity rather than neuronal loss.

Collectively, these studies establish that α-, β-, and γ-synucleins act cooperatively to preserve presynaptic integrity by supporting SNARE-complex assembly and maintaining synaptic organization. Deletion of synucleins does not impair early neuronal development but leads to progressive, age-dependent decline in synaptic transmission and neurological function. Collectively, these findings identify synucleins as important molecular regulators that support the long-term stability and functional efficiency of neuronal communication. By maintaining synaptic integrity over time, synucleins appear essential for preserving normal neurotransmission during aging. Consequently, disruption of their physiological function may compromise synaptic homeostasis and contribute to cellular mechanisms that underlie neurodegenerative disorders, including PD.

## When function turns fatal: aggregated α-synuclein derails vesicle dynamics

**Figure 6:**
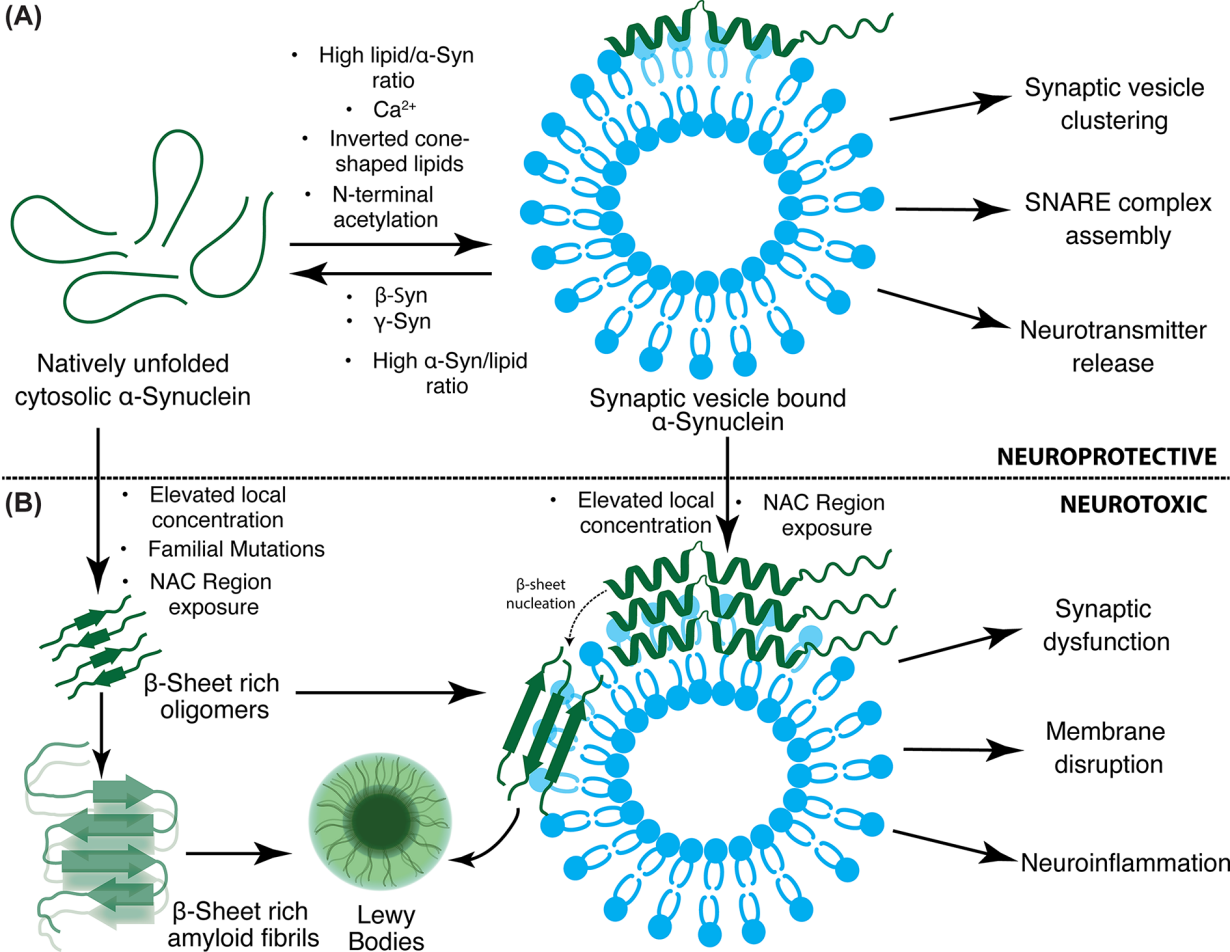
Dual conformational pathways of α-Synuclein: neuroprotective versus neurotoxic (**A**) α-Syn resides in a dynamic equilibrium between a vesicle-associated, α-helical conformation and an intrinsically disordered cytosolic state. The membrane-bound form is essential for SV clustering, SNARE complex assembly, and efficient neurotransmitter release. Multiple biophysical and lipid-dependent factors promote the transition from the natively unfolded monomer to the membrane-engaged α-helical state. (**B**) The intrinsically disordered α-Syn monomer transitions into toxic, β-sheet-rich amyloid fibrils through the formation of oligomeric intermediates. These oligomers act as nucleation seeds capable of templating fibrillization, even for the α-helical, membrane-bound pool. Emerging evidence further indicates that vesicle-associated α-Syn can initiate amyloid formation under conditions of elevated protein concentration with increased NAC-region exposure. Across these pathways, the accumulation of amyloid fibrils drives synaptic dysfunction and contributes to the progressive loss of dopaminergic neurons.

α-Syn exists in a dynamic equilibrium between a membrane-associated, α-helical conformation that supports its normal physiological roles and a predominantly disordered form that resides in the cytosol (Figure 6A) [116]. The membrane-cytosol balance of α-Syn is essential to its function and pathology, yet the role of membrane binding in disease remains unclear. When monomeric α-Syn associates with membranes, it is typically stabilized in an α-helical state that resists aggregation (Figure 6A), whereas the soluble, unbound population exhibits a markedly greater propensity to misfold (Figure 6B) [117]. Aggregation of α-Syn is a hallmark of synucleinopathies, most notably PD [12,118], DLB [119,120], and MSA [121,122]. In these conditions, α-Syn misfolds and builds up as oligomers, protofibrils, and eventually fibrils that form LBs or LNs (Figure 6B) [12,15,123]. These abnormal species can seed further misfolding, creating a self-reinforcing cycle that reduces the pool of functional protein and disrupts normal SV activity. Because α-Syn is important for vesicle maintenance, trafficking, and neurotransmitter release, its aggregation directly impairs these processes and contributes to disease progression [118,124].

A variety of cellular perturbations can shift the delicate balance between membrane-anchored α-Syn and its freely diffusing cytosolic pool, although the mechanisms underlying this equilibrium remain actively debated (Figure 6). Changes in SV curvature, alterations in lipid composition, and disruptions in proteolipid organization can weaken membrane affinity and expand the soluble fraction [29,125]. In this context, aging has been shown to remodel SV organization through coordinated alterations in both protein composition and membrane lipid architecture, as revealed by recent multi-omics studies [126]. Earlier proteomic evidence from Jiang et al. demonstrated that synaptic lipid rafts from aged brain tissue exhibit a broad decline in key vesicle-associated proteins [127]. Aged lipid rafts exhibited decreased abundance of synapsins, septin 5, syntaxin-binding protein 1, and cytoskeletal proteins. Extending these findings, Virginia Gao et al. integrated proteomic and lipidomic analyses to demonstrate that aging is not merely associated with protein loss but instead involves coordinated remodeling of SV molecular architecture [126]. Their findings highlight selective alterations in vesicle-associated proteins involved in trafficking and fusion, including synaptotagmins, synapsins, SNARE regulators, and Rab GTPases, alongside significant lipid compositional shifts characterized by increased lysophospholipids and altered PS, PC, and PE species with greater unsaturation. These lipid changes are expected to influence membrane curvature, fluidity, and charge, thereby modulating protein-lipid interactions critical for vesicle dynamics. Collectively, these findings suggest that aging induces a progressive, multi-layered reorganization of SVs, ultimately compromising synaptic efficiency.

Genetic mutations linked to PD [128,129], oxidative modification of membrane lipids [130], and crowding-induced competition at vesicle surfaces further destabilize the protective α-helical, membrane-bound state. Ten out of the 12 familial point mutations reported to date are present in the N-terminal region: G14R [131], V15A [132], A30G [133], A30P [134], E46K [135], H50Q [136], G51D [137], A53E [138], A53T [139], and A53V [140]. Several studies have examined the membrane-binding properties of these α-Syn mutants; however, the model membranes employed often do not faithfully reproduce the compositional complexity and biophysical properties of SVs. Mutations such as A30P and A53T map to the imperfect repeat region of α-Syn, a domain critical for membrane engagement. Consistent with this, the A30P variant exhibits a marked reduction in vesicle binding, whereas the A53T mutant largely retains membrane affinity, displaying binding behavior comparable to that of the wild-type protein [141, 142, 143]. These mutation-specific differences influence α-Syn conformation, vesicle interactions, and aggregation behavior, thereby highlighting the conformational and pathogenic heterogeneity among PD-linked α-Syn variants. The resulting increase in the cytosolic pool of unbound, monomeric α-Syn enhances its propensity for aggregation. However, the conformational transition from a random-coil state to cross-β-sheet-rich assemblies is further influenced by a range of additional cellular and biochemical factors [15,117]. Additionally, aging may influence α-Syn conformation through increased oxidative stress and progressive impairment of protein clearance pathways. Elevated oxidative stress during aging promotes oxidative modifications of α-Syn, destabilizing its native conformational equilibrium and favoring the formation of misfolded oligomeric and β-sheet-rich assemblies [144,145]. In parallel, aging-associated decline in protein homeostasis mechanisms, particularly autophagy and lysosomal degradation pathways, reduces the efficient clearance of aberrant α-Syn species, thereby facilitating their accumulation and aggregation [146–148]. Together, these processes shift α-Syn from its physiological conformational states toward pathogenic aggregation-prone conformations associated with synucleinopathies.

A recent study by Carnazza et al. showed that although α-, β-, and γ-synucleins are all abundant in neurons, β- and γ-synucleins bind SV membranes far more weakly than α-Syn [149]. Using liposome-binding assays, circular dichroism, FRET, and brain-fractionation approaches, the authors demonstrate that β- and γ-synucleins can form heteromeric complexes with α-Syn, reducing its affinity for SVs in a dose-dependent manner. This interaction shifts α-Syn from the membrane-bound pool toward a more cytosolic population (Figure 6A). The findings suggest that β- and γ-synucleins act as endogenous regulators of α-Syn’s presynaptic roles by tuning its vesicle association, a mechanism that may also influence its propensity to aggregate under pathological conditions [149].

In parallel, β-synuclein competes for acidic phospholipid sites on vesicles, regulating α-Syn surface density and preventing aberrant clustering. γ-synuclein, while binding membranes more transiently, influences α-Syn’s membrane residence time and vesicle pool organization [149]. Together, these homologues sustain presynaptic proteolipid homeostasis, fine-tuning vesicle clustering and neurotransmitter release while safeguarding against pathological aggregation. Collectively, these factors may bias the system toward a more aggregation-prone unbound population, a transition thought to initiate early pathogenic events, yet the relative contribution of each perturbation and whether membrane disengagement is causal or consequential remains a subject of ongoing debate.

Binding to lipid membranes has also been shown to accelerate the formation of pathogenic α-Syn assemblies [31,32,150]. Roeters and colleagues combine interface-specific vibrational spectroscopy with molecular dynamics simulations to delineate how monomeric α-Syn interacts with anionic lipid membranes [34]. At low or physiological concentrations, α-Syn exists in a flat, surface-aligned helical conformation. At higher concentrations, the protein shifts to an upright, membrane-protruding orientation in which the aggregation-prone NAC regions come in close proximity of each other [34]. This reorientation promotes lateral interactions between membrane-bound monomers, providing a mechanism for surface-catalyzed nucleation [34]. Overall, the work reveals a concentration-dependent switch in membrane binding that links physiological function to early steps in pathogenic aggregation. Taken together, membrane interaction appears protective under physiological conditions but becomes linked to aggregation only after α-Syn has already undergone pathogenic conformational transitions.

## Conclusion

α-Syn exists in a dynamic equilibrium between a membrane-bound α-helical conformation and a cytosolic intrinsically disordered ensemble, and this balance underpins its diverse physiological and pathological behaviors. Its ability to reversibly adopt α-helical structure on highly curved SVs allows it to cluster vesicles, scaffold SNARE complex assembly, and fine-tune neurotransmitter release.

At a physiological level, α-Syn functions as a nanoscale ‘shape reader,’ translating the geometric and compositional features of lipid membranes into functional outcomes. Its N-terminal amphipathic helices are exquisitely tuned to recognize curvature, binding preferentially to small, highly curved vesicles and adopting a bent helical conformation matching the convex membrane surface. The interplay between cone-shaped and inverted cone-shaped lipids creates a membrane landscape that empowers α-Syn to serve simultaneously as a curvature sensor and a mechanical modulator of the vesicle cycle. This curvature-dependent binding is not passive: it directs vesicle clustering, docking, and priming, stabilizes vesicles near active zones, and promotes efficient assembly of the SNARE fusion machinery [9]. Importantly, these interactions remain low-affinity and reversible, ensuring compatibility with rapid synaptic turnover. This same structural plasticity, however, also introduces vulnerability: subtle shifts in lipid composition, membrane curvature, or α-Syn concentration can expose the aggregation-prone NAC domain, converting a physiological membrane sensor into a nucleation seed for oligomers and fibrils [33,34,130].

A growing body of evidence indicates that SVs create a highly tuned nanoscale environment in which membrane curvature, lipid packing defects, and local macromolecular crowding jointly determine α-Syn’s conformational preferences [126]. When these membrane features deteriorate through aging, oxidative lipid remodeling, or disease-associated mutations, the equilibrium shifts toward cytosolic monomeric α-Syn, expanding the pool of disordered species capable of initiating primary nucleation (Figure 6). Vesicle-associated proteins such as VAMP2, synapsin, and Rab3a normally preserve α-Syn in its functional membrane-bound regime through multivalent, low-affinity interactions that support vesicle clustering while suppressing aberrant self-assembly [11,106–108]. Conversely, familial mutations, oxidative lipid damage, and competitive displacement by β- and γ-synucleins destabilize this protective nanoscale architecture [149]. Notably, several studies also suggest that early misfolding events can occur directly on membrane surfaces, indicating that both membrane-bound and cytosolic routes may contribute to pathogenic initiation. This paradox underscores the central theme that α-Syn's pathogenicity arises not from a gain of aberrant structure alone but from the loss of finely tuned lipid-protein homeostasis.

Once misfolding begins, α-Syn oligomers interfere with vesicle trafficking, disrupt SNARE-mediated fusion, and dampen Ca^2+^-evoked exocytosis, driving the synaptic dysfunction characteristic of PD and related synucleinopathies. Together, these findings underscore that the molecular fate of α-Syn is tightly governed by its membrane environment and that disturbances in this equilibrium at the level of curvature, lipid chemistry, or protein competition represent critical inflection points at which normal presynaptic physiology can transition toward early neurodegeneration.

## Future directions

Despite major progress, several fundamental questions remain unresolved. How the nanoscale organization of lipid microdomains and curvature gradients across the presynaptic terminal shapes α-Syn’s binding modes in living neurons is still unclear. Equally critical is defining the mechanisms that govern the shift from α-helical membrane association to early oligomerization and determining whether these transitions are reversible *in vivo*. The physiological and stress-responsive roles of β- and γ-synucleins as modulators of α-Syn membrane residency remain incompletely understood, particularly in the context of aging, metabolic stress, or neuroinflammation.

Rapid advances in structural and chemical imaging now make these questions experimentally tractable. Cryo-electron tomography, native mass spectrometry, and high-resolution live-cell imaging are beginning to visualize α-Syn’s conformational ensemble directly on native vesicle membranes, offering the possibility of capturing the earliest pathogenic intermediates *in situ*. Complementary approaches such as lipidomics and spatially resolved biophysical mapping of neuronal membranes promise to reveal how disease-associated perturbations including altered cholesterol content, phospholipid asymmetry, or the presence of familial α-Syn mutations influence α-Syn’s binding landscape. At the molecular scale, single-molecule approaches provide critical resolution into the structural heterogeneity and dynamic interaction profiles of intrinsically disordered regions, revealing transient states obscured in ensemble measurements [151]. Together, these convergent methodologies offer a multiscale framework for understanding why specific neuronal populations, including dopaminergic neurons of the substantia nigra, display exceptional vulnerability to α-Syn pathology.

A deeper integration of membrane biophysics, synaptic proteomics, and cellular physiology will be essential to understand how α-Syn transitions from a presynaptic organizer to a driver of synaptic failure. Ultimately, strategies that stabilize membrane-tethered conformations or prevent the concentration-dependent redistribution of membrane-bound monomers into the cytosolic phase may provide new therapeutic avenues to halt synucleinopathies at their earliest molecular inflection point.

In summary, α-Syn biology cannot be separated from its membrane context. Its dual identity as both a chaperone of SV dynamics and a trigger of neurodegenerative pathology emerges from the same structural motifs that sense, adapt to, and remodel curved lipid environments. Deciphering this proteolipid interplay represents a central frontier in neurobiology, where understanding curvature, composition, and molecular disorder at the nanoscale may ultimately yield macroscopic solutions to human disease.

## Abbreviations

α-Syn: α-Synuclein
LBs: Lewy bodies
LNs: Lewy neurites
LysoPC: lysophosphatidylcholine
MSA: multiple system atrophy
PC: phosphatidylcholine
PD: Parkinson’s disease
PE: phosphatidylethanolamine
PI: phosphatidylinositol
PS: phosphatidylserine
PTMs: post-translational modifications
SNARE: soluble N-ethylmaleimide-sensitive factor attachment protein receptor
SV: synaptic vesicle
VAMP2: vesicle-associated membrane protein 2
